# Papain associated with root planing in treatment of periodontitis

**DOI:** 10.1590/0103-644020255813

**Published:** 2025-04-07

**Authors:** Luiz Eduardo Monteiro Dias da Rocha, Ricardo Guimarães Fischer

**Affiliations:** 1 Department of Periodontology, Rio de Janeiro State University, Rio de Janeiro, Brazil.

**Keywords:** periodontitis, papain, subgingival scaling, root planning, dental calculus

## Abstract

Although subgingival scaling and root planing (SRP) are the “gold standard” treatment for periodontitis, these processes may remove excessive cement, exposing dentin and causing hypersensitivity, or producing defects such as ridges and grooves, leaving residual calculus and preventing access to the whole of the root surface. A papain gel that removes carious dentin (Papacárie®) has been introduced and this gel may also help to remove supra and subgingival calculus, acting on its organic portion and biofilm, decreasing the consumption of cement and generating lower levels of aerosol compared to SRP. The aim of the present split-mouth, single-blind study was thus to compare the effectiveness of papain gel with subgingival root planning (RP). After receiving oral hygiene instruction, supragingival scaling, and coronary polishing, 18 periodontitis patients (6 women and 12 men, mean age of 51 ± 8 years) were included in the study. Treatment involved subgingival application of the gel for one minute, followed by RP while the control group received subgingival SRP. Treatment was conducted by three operators and a blinded examiner recorded the following clinical parameters at baseline and after three months: plaque index (PI), bleeding on probing (BoP), probing pocket depth (PPD), clinical attachment level (CAL), and gingival recession (GR). The results showed significant improvements in clinical parameters, in both the treatment and the control group, with no statistical difference between them. It was concluded that papain did not provide any additional

## Resumo

Apesar da raspagem e alisamento radiculares (RAR) constituírem o “padrão ouro” para o tratamento da periodontite, estes procedimentos podem remover cemento em excesso, expondo a dentina e causando hipersensibilidade ou produzindo defeitos como sulcos e ranhuras, deixando cálculo residual e impedindo o acesso a toda a superfície radicular. Um gel de papaína que remove a dentina cariada (Papacárie®) foi desenvolvido e este gel pode auxiliar também na remoção do cálculo supra e subgingival, agindo na sua parte orgânica e no biofilme, com redução de remoção de cemento e gerando níveis mais baixos de aerossóis quando comparados com a RAR. O objetivo do presente estudo em boca-dividida, duplo-cego, foi comparar a eficácia ou não do gel de papaína associado ao alisamento radicular (AR). Após receber instrução de higiene oral, raspagem supragengival e polimento coronário, 18 pacientes com periodontite (6 mulheres e 12 homens, média de idade de 51 ± 8 anos) foram incluídos no estudo. O tratamento consistiu em uma aplicação de gel subgengival por um minuto, seguido de AR, enquanto o grupo-controle recebeu RAR. O tratamento foi conduzido por 3 operadores e um examinador registrou os seguintes parâmetros clínicos na fase inicial e após 3 meses: índice de placa, sangramento à sondagem, profundidade de bolsa à sondagem, nível de inserção clínica e recessão gengival. Os resultados demonstraram melhoras significantes nos parâmetros clínicos tanto no grupo-teste como no grupo-controle sem diferença estatística entre ambos. Concluiu-se que a papaína não trouxe benefícios à RAR.

**Figure:**
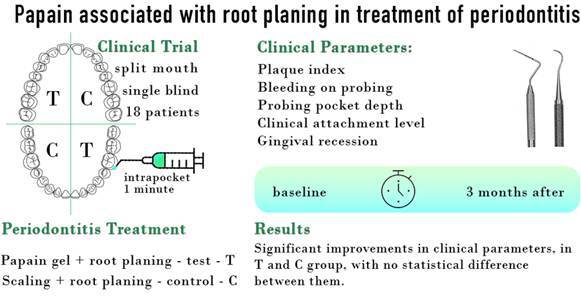

## Introduction

Periodontitis is a major public health problem due to its high prevalence, and since it may lead to tooth loss and disability, it negatively affects chewing function and aesthetics, is a source of social inequality, and significantly impairs quality of life. Severe periodontitis affects about 19% of people aged greater than 15 years, representing more than one billion cases worldwide. It is a chronic multifactorial inflammatory disease associated with dysbiotic dental plaque biofilms and a complex opportunistic infection modified by the host inflammatory response ^1^ ^,^ ^2^ ^,^ ^3^. The most effective and recommended treatment involves anti-infective therapy. This approach relies on adequate patient plaque removal through good oral hygiene practices and mechanical debridement by an oral health professional. Even today, scaling and root planning (SRP) remains an essential part of successful periodontal therapy ^2^. However, SRP are difficult procedures to perform, requiring professional skill and training ^4^. It is accepted to be the basis for all periodontal therapy, and any additional therapies should be considered adjunctive and supplemental ^1^. The collective evidence from numerous clinical trials reveals a consistency of clinical response in the treatment of periodontitis by SRP using manual, sonic, or ultrasonic instrumentation. Thus, SRP remains the 'gold standard' to which more recently developed therapeutic modalities must be compared. Inherent to the clinical evaluation of SRP are such concerns as manual versus sonic and ultrasonic instrumentation, control of subgingival bacterial populations, removal of calculus, root smoothness, and changes in various clinical parameters, e.g., probing depth, attachment levels, bleeding on probing and gingival inflammation ^2^. Certain procedures generate aerosols and droplets and pose a risk of respiratory infection by transmission of SARS-CoV-2 and other diseases. Ultrasonic scaling poses a high risk of contamination risk while that of SRP is low ^5^. 

In recent years, a papain-based gel (Papacárie®, São Paulo, SP, Brazil) has been used for the removal of dental caries, the softening of infected dentin, and the preservation of healthy tissue ^6^. Papain is an enzyme with strong proteolytic properties whose use has been widespread in the food, pharmaceutical, and cosmetics industries ^6^. Proteolytic enzymes are widely used for various medical purposes such as debridement, as well as the removal of necrotic and infected tissues in wounds or burns. Among them, papain (EC 3.4.22.2), an endolytic cysteine protease enzyme from the papaya (Carica papaya L.) latex, is nowadays offered as an anti-inflammatory, anti-coagulant, and hemolytic agent, also facilitating debridement, and speeding up tissue recovery. In addition, papain was reported as an anti-biofilm ^7^, anti-plaque, and anti-gingivitis agent ^8^. The removal of supragingival and subgingival calculus could, therefore, be facilitated by the use of a papain-based gel due to its proteolytic action on the organic part of the dental calculus. The aim of the present study was thus to analyze the efficacy of a papain-based gel in combination with only RP in the subgingival region, as compared to SRP alone.

## Materials and methods

**Figure 1:**
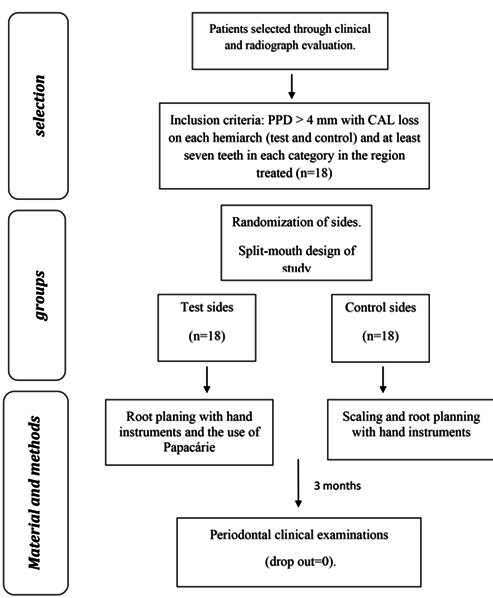
Flow diagram

This was a split-mouth, single-blind study. Randomization was performed to choose the test and control hemiarches by draw carried out by each operator (JM, MR, CR). The examiner (LEMDR) was blind to the non-surgical procedure performed. If the superior right hemiarch was treated using the test protocol, the left side received the control treatment and vice versa in the inferior arch. The sequence of selection, treatment and evaluation can be seen in Figure 1.

### Sample selection

A sample test was conducted for the minimum difference of 1.2, and the result was 14 patients, with an 80% confidence interval and a 5% possibility of error.

Eighteen patients were selected (6 women and 12 men, mean age of 51 ± 8 years) - because some could be lost during the 3 months of the study - with periodontitis were selected at Dentistry School of University of Rio de Janeiro, Brazil. No patient dropped out of the research.

The inclusion criteria were: ^1^ not having received periodontal treatment for at least six months before commencement of the experiment, ^2^ not having received anti-inflammatory or antibiotic therapy for at least two months, ^3^ presenting with at least four sites with PPD > 4 mm with CAL loss on the hemiarch test(s) and hemiarch control(s) and at least seven teeth in each category in the region treated. Three patients were smokers and two were found to be diabetic during treatment. All signed a "consent form" and the project was approved by the Ethics Committee of the Pedro Ernesto University Hospital of the State University of Rio de Janeiro (no. 2141-CEP/HUPE), and was conducted in accordance with the Helsinki Declaration of 1975, as revised in 2013. This study was submitted to the Brazilian clinical trials (RBR-6cvpj4g).

### Periodontal Assessment of Patients

Three operators (periodontal specialists) performed all clinical procedures. The first operator (JM) treated seven patients, the second (MR) eight, and the third (CM) three. All patients were examined at the baseline and after three months by the same blinded examiner (LEMDR). The intra-examiner reproducibility test was performed by the examiner (LEMDR) in four patients, with an interval of one week between each examination and a total of 1188 sites. The level of agreement was 93.5% for a ±1mm difference between PPD and CAL measurements.

Prior to the experiment, each patient received appropriate oral hygiene instructions, supragingival scaling, and dental prophylaxis using a rubber cup and prophylactic paste (Herjos F®, Vigodent, Rio de Janeiro, Brazil). The baseline examination was performed one week later and covered the following clinical parameters: plaque index (PI) (Silness and Loe, 1964), modified by the use of a dye (Replak® liquid, Dentisply, Rio de Janeiro, Brazil); bleeding on probing (BoP); PPD, CAL and GR. PI and BoP were measured at four sites (mesial, vestibular, distal, and lingual) while PPD, CAL, and GR were measured at six sites (mesiovestibular, vestibular, distovestibular, mesiolingual, lingual, and distolingual). Clinical measurements were taken using a calibrated periodontal probe (CP-15 University of North Carolina PCPUNC156, Hu-Friedy®, Chicago, USA). On the control side, SRP was carried out using Gracey 3-4, 5-6, 9-10, 11-12 13-14 and McCall 17-18 curettes (Hu-Friedy®, Chicago, USA). The procedure was performed under local anesthesia, by region. SRP was performed until a surface was considered free of biofilm and calculus by the operator. On the test side, the papain-based gel (Papacárie®, Fórmula e Ação, São Paulo, Brazil) was meticulously applied to the subgingival region using a syringe and needle without a tip, ensuring it completely filled the subgingival pocket area for a duration of 60 seconds. RP was subsequently performed using the same curettes for the whole root surface. After the procedure, the subgingival region was irrigated with distilled water. Papacárie® has a formula composed of papain, chloramine, toluidine blue, preservatives, thickeners, stabilizers, and deionized water - registered with ANVISA (National Health Surveillance Agency) under the number 80013980025. When patients did not have the four appropriate hemiarches needed for the research, treatment was performed in two hemiarches. The instrumentation time was measured for both the test and the control sides. Patients received further advice on oral hygiene and supragingival scaling methods, if necessary, after 28 days and after three months. The time taken to perform each procedure, SRP and gel+RP, was measured for each patient. The study was conducted for a period of 2 years and 6 months between sample selection, treatment, and follow-up after 3 months.

Statistical data analysis was carried out using the parametric ANOVA and t-tests on the SPSS 23.0 statistics software package. The data were tested for normality using the Shapiro-Wilk Test and the distribution of the sample was confirmed to be normal. Differences were statistically significant when p≤0.05.

## Results

There was no statistically significant difference in the PI between the test and the control sides at the baseline. There was, however, an increase in the quantity of plaque (index 2-3) on both sides throughout the study (table 1).

The mean % of sites presenting with BoP was significantly lower after 3 months (table 1). The mean number of sites showing a reduction ≥2mm in PPD was similar in both groups. The mean percentage of PPD≥4 mm in both groups was found to have been significantly reduced after three months (Table 1). There was no statistically significant difference between the test and the control sides (table 1). The mean percentage of sites with CAL gain ≥2 mm after three months was 11.8% on the test side and 12.3% on the control side (p <0.05) (Table 1).

On follow-up, measurements revealed a greater reduction in PPD ≥7 mm. The greatest reduction in PPD was observed on the test side, with a mean decrease from 7.5 mm at the baseline to 5.6 mm after three months (p <0.05). In the control, however, PPD was reduced from 8.1 mm to 6.4mm over the same period (p <0.05). There was no statistically significant difference between both groups (Table 1).

There was no variation in shallow pockets on either the test or the control sides. Sites with initial PPD 4-6 mm had a CAL significant gain of 0.6mm and 0.8mm on the test and control sides respectively after three months. In deep sites, the highest CAL gain was found on the test side (1.5 mm), while on the control side, there was a 1.2 mm CAL gain after a period of three months (p <0.05), but without significant difference between the groups (Table 1).

An increase in GR was observed in both the test and the control sides. The greatest difference was observed in pockets with an initial PPD ≥7 mm (Table 1).

**Table 1:** Mean (±SD) percentage of plaque index (Pi) 2-3, bleeding on probing (BoP), probing pocket depth (PPD) ≥4 mm, percentage of sites of clinical attachment level (CAL) ≥2 mm and mean (±SD) of probing depth, clinical attachment level and gingival recession (GR) 1 to 3 mm, 4 to 6mm and ≥7 mm.

Clinical	Baseline		3 months	
Parameters	Test	Control	Test	Control
PI 2-3 (%)	54.0 (±21.8)	57.4 (±21.0)	65.6* (±17.4)	66.9 (±17.1)
BoP (%)	41.6 (±18.4)	40.1 (±20.8)	19.1* (13.6)	21.0* (±14.8)
PPD ≥4mm (%)	46.1 (±11.5)	49.4 (±12.6)	31.7* (±15.7)	35.2* (±15.6)
PPD 1 to 3mm	2.4 (±0.4)	2.4 (±0.3)	2.3 (±0.3)	2.3 (±0.3)
PPD 4 to 6mm	4.8 (±0.4)	4.9 (±0.4)	4.0* (±0.7)	4.0* (±0.7)
PPD ≥7mm	7.5 (±0.5)	8.1 (±0.8)	5.6* (±1.5)	6.4* (±1.6)
CAL ≥2mm (%)			11.8* (±7.1)	12.3* (±6.0)
CAL 1 to 3mm	3.2 (±0.7)	3.1 (±0.7)	3.2 (±0.7)	3.1 (±0.7)
CAL 4 to 6mm	5.5 (±0.8)	5.5 (±0.6)	4.9* (±1.4)	4.7* (±1.0)
CAL ≥7mm	8.2 (±1.1)	8.8 (±1.0)	6.7* (±2.0)	7.7* (±2.0)
GR 1 to 3mm	0.8 (±0.7)	0.8 (±0.6)	0.9 (±0.8)	0.8 (±0.6)
GR 4 to 6mm	0.7 (±0.8)	0.6 (±0.5)	1.0* (±0.9)	0.7* (±0.6)
GR ≥7mm	0.7 (±1.2)	0.7 (±1.1)	1.1* (±1.2)	1.3* (±1.6)

*significant to the baseline.

The average treatment time per treated tooth was 9min and 15sec for the test side and 8min for the control. The instrumentation time was only significantly longer for operator 1 in the test treatment (difference of 2.1 minutes), which did not occur with the other operators (Table 2).

**Table 2:** Meantime (±SD) spent per tooth (minutes) to perform the test and control treatment by each operator and mean (M) of the total time per tooth of all patients.

	Test	Control	n	p
Operator 1	10.6 (±1.4)	8.5 (±1.8)	7	0,03
Operator 2	8.8 (±2.1)	8.7 (±2.1)	8	0,78
Operator 3	8.7 (±1.1)	5.2 (±0.5)	3	0,11
M per tooth	9.2 (±2.2)	8 (±2.2)	18	0,06

## Discussion

The present study aimed to verify the efficacy of non-surgical periodontal treatment involving the application of a papain-based gel in the subgingival region in patients with periodontitis. In the present study, the average proportion of sites with CAL ≥2 mm was 11.8% on the test side and 12.3% on the control side for the 3 months. In both cases, there were statistically significant changes in the baseline. Sites with initial PPD equal to 4 to 6mm showed a CAL gain of 0.6 and 0.8 mm for the test and the control sides, respectively. Sites with an initial PPD ≥7 mm presented a mean CAL gain of 1.5 mm for the test side and 1.1 mm for the control side within a period of three months. There was, however, no statistical difference between the two forms of treatment.

No study previously used Papacarie^®^ associated with root planning. Studies were used to facilitate caries removal ^6^. The relevance of this study is to describe a new procedure in the treatment of periodontitis as it probably removes less tissue compared to SRP (on the control side) because treatment was restricted to RP (no scaling). Although there is no study related to periodontal treatment, the use of Papacarie^®^ removes necrotic dentine in cariology ^6^.

The results observed in the present study concur with those of several studies that have compared the effect of surgical and non-surgical treatment in patients with moderate to advanced periodontitis ^9^ ^,^ ^10^ ^,^ ^11^. Cobb ^12^ examined numerous studies of non-surgical periodontal therapy, using manual and ultrasonic instruments and found an average reduction in PPD of 1.3 mm for 6 mm pockets and 2.2 mm for pockets ≥7 mm. A systematic review found a weighted mean reduction of 0.6 mm for pockets that had initially been ≥5 mm ^10^. Clinical evidence suggested a mean PPD reduction of 1.7 mm and 2.6 mm for deep sites (>6 mm) ^2^.

In the present study, the proportion of PPD ≥4 mm compared to the total sites significantly decreased on both sides after three months of treatment. On the test side, the reduction was from 46.1% to 31.7%; on the control side, from 49.4% to 32.5%. The results for PPD showed that treatment was effective and there was no statistical difference between sides. A reduction in PPD is the result of two phenomena: (i) contraction of the soft tissue wall of the pocket, taking the form of recession of the gingival margin, caused by decreased inflammation and edema in the soft tissue; and (ii) CAL gain ^9^ ^,^ ^13^. The evidence suggested that a mean proportion of closed pockets of 74% ^2^.

No CAL changes were observed 3 months after treatment for shallow sites in this study. Pihlstrom et al. ^(^ ^14^ observed loss of attachment in shallow sites (1-3 mm) in four years of observation. In 4-6 mm sites, the mean CAL gain was 0.6 mm in six months and ≥7 mm sites showed a gain of 1.4 mm in the same period. In this study, the corresponding values were 0.6/0.8 mm for 4-6 mm sites and 1.5/1.1 mm for ≥7 mm sites for test and control, respectively. Systematic reviews have indicated that CAL may improve by 0.5-2 mm ^10^ ^,^ ^11^.

In the present study, gingival recession tended to increase significantly after treatment, with a greater difference in the case of initially deep sites, with 0.4 mm on the test side and 0.6 mm on the control side. The GR for sites with an initial PPD of 4-6 mm was lower (p<0.05). However, this figure was smaller than the one recorded by another study, which reported an increase of 0.6 mm for shallow sites, 1.1 mm for moderate sites, and 1.5 mm for deep sites, occurring mainly in the first month after treatment ^15^.

The mean proportion of sites with PI was 56.3% at the baseline and 66.6% after three months. There was a statistically significant difference between the test and the control sides, and also between the rate on the test side after three months compared to the baseline. Despite this poor performance in terms of plaque control, there was a reduction in PPD, CAL gain or maintenance, and reduction of inflammation, in accordance with a study with a four-year follow-up period ^14^.

A decrease in gingival inflammation occurred, even though the PI remained high. BoP improved throughout the study, with a statistically significant reduction in this parameter from 41.6% to 19.1% and 40.1% to 21% for test and control sides, respectively. This reduction in BoP corroborates the findings of numerous studies that have reported decreased bleeding after plaque control and periodontal treatment (14;15;16). After subgingival instrumentation, a 63% reduction in BoP is expected ^2^.

The average treatment time per treated tooth on the test side was 9.2 min., while on the control side, it was 8.3 min. This difference of approximately one minute may be caused by the time of application and the gel remaining in the subgingival region on irrigation with distilled water after the RP procedure. All operators required a longer period to perform the test procedure. However, the difference between Operator 2, who treated the largest number of patients (n=8), and Operator 1, was only 8 seconds. The mean total instrumentation time per tooth was similar to that reported by Sherman et al. ^15^ (9.4 min.), representing a combination of 3.6 min. of ultrasonic instrumentation and 5.8 min. using manual instruments.

In clinical trials, the time spent per tooth is 10 minutes on average, when performed by trained operators ^17^. The limitation of the study was: that as the methodology was strictly followed, the gel can only be applied once for 1 minute, without being able to be repeated in areas with a greater amount of calculus or residual calculus perceived by the operator. These operators were periodontal specialists in and reported that the test treatment was easier to perform but that only one application of the gel for 1min. in the subgingival region was sufficient in only "some" of the patients.This may be associated with the amount of calculus present and the degree of inflammation at each site, as local bleeding can expel the gel from the area. Stoltze & Stellfeld ^18^, evaluating the permanence of a metronidazole gel applied with a syringe in the subgingival region of the periodontal pocket, found that 60% was immediately lost, with only 40% remaining in place.

It is not clear how much cement should be removed to ensure effective treatment ^19^. The aim of subgingival removal of this calcified tissue is to eliminate calculus, microorganisms, and bacterial endotoxins. However, calculus may already be incorporated into the calcified tissue, thereby causing the cement to be removed in excess, with possible consequent exposure to dentin ^9^. Kepic et al. ^20^ concluded that complete removal of calculus on root surfaces affected periodontal disease is rare, even after two phases of SRP with the use of manual and ultrasonic instruments. The first phase involved a 'closed' approach, and the second phase, conducted after eight weeks, involved flap elevation to provide maximum access and visibility for instrumentation.It should be pointed out that patients with periodontitis may undergo many SRP sessions in the course of treatment and many periodontal maintenance procedures in their lifetime ^21^. The goal of removing all calculus and contaminated cement would thus seem to be unrealistic and probably unnecessary ^19^. Nyman et al. ^22^ surgically treated patients with moderate and advanced periodontitis using a split-mouth model and did not find any differences when SRP was performed meticulously to achieve total removal of calculus, soft deposits, and cement, or when the surface alone was polished with rubber tips to eliminate biofilm after 24 months of observation. 

The present study suggests that effective periodontal therapy can be carried out by way of disaggregation of the subgingival biofilm after the application of a papain-based gel (Papacárie®), in combination with RP. This may reduce viable bacterial colony count, and remove subgingival calculus in such a way as to cause less wear to cement and dentin and less trauma to hard and soft tissues ^23^. This procedure requires a lower degree of skill on the part of the clinical operator, since root scaling is a difficult procedure to perform, especially when periodontal pockets exceed 5mm in depth ^24^. The use of gel may help to perform instrumentation in regions that are difficult to access, such as furcation areas, deep pockets, concavities, and grooves. Further studies, however, are needed to confirm these hypotheses.

## Conclusion

It was concluded that papain did not provide any additional benefit to SRP.
